# Conducting rapid qualitative interview research during the COVID-19 pandemic—Reflections on methodological choices

**DOI:** 10.3389/fsoc.2022.953872

**Published:** 2022-08-11

**Authors:** Marta Wanat, Aleksandra J. Borek, Caitlin Pilbeam, Sibyl Anthierens, Sarah Tonkin-Crine

**Affiliations:** ^1^Nuffield Department of Primary Care Health Sciences, University of Oxford, Oxford, United Kingdom; ^2^Department of Family Medicine and Population Health, University of Antwerp, Antwerp, Belgium; ^3^National Institute for Health Research Health Protection Research Unit in Healthcare Associated Infections and Antimicrobial Resistance, Oxford, United Kingdom

**Keywords:** qualitative, rapid, methodology, COVID-19, healthcare emergency

## Abstract

As the COVID-19 pandemic has shown, setting up studies in time to gather relevant, real-world data enables researchers to capture current views and experiences, focus on practicalities on the ground, and deliver actionable results. Delivering high quality rapid studies in healthcare poses several challenges even in non-emergency situations. There is an expanding literature discussing benefits and challenges of conducting rapid research, yet there are relatively few examples related to methodological dilemmas and decisions that researchers may face when conducting rapid studies. In rapidly-changing emergency contexts, some of these challenges may be more easily overcome, while others may be unique to the emergency, magnified, or emerge in different ways. In this manuscript, we discuss our reflections and lessons learnt across the research process when conducting rapid qualitative interview studies in the context of a healthcare emergency, focusing on methodological issues. By this we mean the challenging considerations and pragmatic choices we made, and their downstream impacts, that shaped our studies. We draw on our extensive combined experience of delivering several projects during the COVID-19 pandemic in both single and multi-country settings, where we implemented rapid studies, or rapidly adapted an existing study. In the context of these studies, we discuss two main considerations, with a particular focus on the complexities, multiple facets, and trade-offs involved in: (i) team-based approaches to qualitative studies; and (ii) timely and rapid data collection, analysis and dissemination. We contribute a transparent discussion of these issues, describing them, what helped us to deal with them, and which issues have been difficult to overcome. We situate our discussion of arising issues in relation to existing literature, to offer broader recommendations while also identifying gaps in current understandings of how to deal with these methodological challenges. We thus identify key considerations, lessons, and possibilities for researchers implementing rapid studies in healthcare emergencies and beyond. We aim to promote transparency in reporting, assist other researchers in making informed choices, and consequently contribute to the development of the rapid qualitative research.

## Introduction

The field of rapid qualitative research has a long-standing history in social sciences (Vindrola-Padros, 2021a). It has origins in the movement to involve local communities in identifying their own needs (Murray, 1999; McNall and Foster-Fishman, 2007), which then spread to the area of public health and social sciences (Richardson et al., 2021). Rapid research may take many forms (Richardson et al., 2021) and indeed researchers have delineated over 15 distinctive approaches in rapid qualitative research (Vindrola-Padros, 2021a). The diversity in approaches has also been reflected in somewhat heterogeneous definitions, based on the type of rapid approach (e.g., McNall and Foster-Fishman, 2007; Beebe, 2014; Vindrola-Padros, 2021a), with some authors highlighting key differences between them (McNall and Foster-Fishman, 2007). Nevertheless, features that seem to be common (but not essential) across these diverse approaches have been identified, including: rapid timeframes; team-based approach; use of multiple methods; iterative nature (e.g., simultaneous data collection and analysis); and a participatory focus, including engagement with relevant stakeholders to set research priorities and facilitate dissemination of actionable findings (Beebe, 2001; McNall and Foster-Fishman, 2007; Vindrola-Padros, 2021a). Indeed, some have urged researchers to think about these features on a continuum rather than as essential for all rapid qualitative studies (Vindrola-Padros, 2021a). For example, while a team-based approach may be beneficial for some studies, for others it may not be possible or useful (Vindrola-Padros, 2021a). It is also worth noting that, alongside the development of rapid approaches, we have also seen researchers creating rapid techniques with the aim of speeding up the process of data collection (through, e.g., mind-mapping, note-taking, or real time transcription) or analysis (through, e.g., omitting transcription, using voice recognition software for transcription, mind mapping, or direct coding from the audio-recordings) (Vindrola-Padros and Johnson, 2020). These techniques, in contrast to rapid qualitative research approaches, can be also used as part of longer-term studies (Vindrola-Padros and Johnson, 2020).

As qualitative researchers wanted to produce meaningful yet rapid research findings during the COVID-19 pandemic, the use of rapid qualitative research methods has seen an increase. This has been noted previously, with researchers turning to rapid approaches in other pandemics such as Ebola (Johnson and Vindrola-Padros, 2017). The COVID-19 pandemic has thus ignited further interest in rapid qualitative approaches, and created a particular opportunity to move the field forward. Successful setup and implementation of rapid qualitative studies in healthcare pose several challenges even in non-emergency situations. There may be unique challenges to conducting qualitative studies in extraordinary circumstances, such as a pandemic (Graetz et al., 2022). In rapidly-changing emergency contexts, some of these challenges may be novel, magnified, or emerge in different ways and at different stages of the research process, whilst others may be more easily overcome. Understanding these challenges as well as successful ingredients is important. Discussions of such methodological choices are still limited, although they are needed to assist researchers interested in rapid approaches to make informed research design decisions (Vindrola-Padros, 2021a), and there have been calls to compare the reliability of rapid techniques to identify their strengths and weaknesses (Johnson and Vindrola-Padros, 2017; Vindrola-Padros and Johnson, 2020). In this manuscript, we reflect on the methodological decisions and their consequences in the context of implementing rapid qualitative studies. The aim of our discussion is to identify key considerations, lessons, and possibilities for researchers implementing rapid studies in healthcare emergencies and beyond. This offers transparent guidance for researchers to make informed choices, and is an important part of preparedness in responding to pandemics and other urgent healthcare needs.

## Materials and methods

This article draws on our experiences with six studies that we conducted during (and which related to) the COVID-19 pandemic. Throughout the process of data collection, analysis, and writing up these studies (between April 2020 and December 2021), all authors met on a regular basis to discuss their reflections on methodological choices within, and between study teams. These meetings provided the groundwork for this manuscript, as they allowed us to reflect on methodological dilemmas in each study. With time, the meetings enabled cross-study reflections and more theoretically-informed discussions around the suitability and feasibility of using rapid methods in our studies at the time and in the future, leading us to identify key points of comparison and learning. We further interrogated our understandings through repeated rounds of writing and reviewing related manuscripts. As our discussions and meetings continued, we identified a number of challenges and issues. Two issues were particularly important to our studies, which related to two of four commonly described features of rapid qualitative research, namely the process of implementing a team-based approach and ensuring rapid data collection (McNall and Foster-Fishman, 2007; Vindrola-Padros, 2021a). Within these two key features, we identified a number of issues which we kept coming back to and became the focus of this manuscript. We use our six studies to demonstrate to the reader how these methodological choices and challenges have played out. The key features of these studies are summarized in Table 1; in brief:

1. RECOVER-QUAL (Wanat et al., 2021a,b, 2022) was a qualitative study in eight European countries investigating patients' and healthcare professionals' (HCPs') experiences of receiving/delivering care for respiratory symptoms in primary care during 2020.
2. FACTS (Hirst et al., 2021; Wanat et al., 2021c) was a mixed-methods study embedded within a cohort study exploring university students' and staff' experiences of using Lateral Flow Tests for COVID-19.
3. SCIENTIST (Colman et al., 2021) was a qualitative study exploring views and experiences of scientists working on government advisory boards during the COVID-19 pandemic.
4. STEP-UP (Borek et al., 2021) was a qualitative study with primary care HCPs about the impact of COVID-19 on antibiotic prescribing and stewardship, conducted as part of a larger program of research.
5. HCP Policy and Experiences study (Borek et al., 2022; Pilbeam et al., 2022) was a longitudinal qualitative study exploring the dynamics of policy development and HCPs' experiences of working in the COVID-19 pandemic.
6. HOUSEHOLD (Verberk et al., 2021) was a mixed-method study in Belgium and the Netherlands investigating how household members navigated COVID-19 recommendations to prevent the spread of infection within the home.

**Table 1:** Overview of conducted rapid studies.

**Study name**	**RECOVER-QUAL**	**FACTS**	**SCIENTIST**	**STEP-UP**	**HCP Policy and Experiences**	**HOUSEHOLD**
Sample	146 interviews with 66 patients and 80 HCPs, from eight countries	18 interviews with 10 university staff members and eight students	21 interviews with scientists from five countries	24 interviews with 18 HCPs	105 interviews with 14 HCPs	18 interviews with household members from two countries
Design	Stand-alone qualitative study	Mixed-methods study embedded within a cohort study	Stand-alone qualitative study	Longitudinal qualitative study (cross-sectional—most participants were interviewed twice), adapted from a standard pre-planned study	Longitudinal qualitative study (trajectory—participants were interviewed multiple times over a year)	Mixed-methods study
Data collection period	April to July 2020	December 2020 to January 2021	December 2020 to April 2021	Two time-points: November 2020, and May 2021	February 2020 to February 2021	May to July 2020
Setting and countries	England, Belgium, the Netherlands, Ireland, Greece, Poland, Sweden and Germany	England	England, Belgium, the Netherlands, Sweden and Germany	England	England and Scotland	Belgium and the Netherlands
Data collection methods and techniques	Semi-structured interviews	Semi-structured interviews	Semi-structured interviews	Semi-structured interviews	Semi-structured interviews	Semi-structured interviews
Transcription	Fully transcribed	Not transcribed	Fully transcribed	Fully transcribed	Partially transcribed	Not transcribed
Analysis	Deductive and inductive thematic analysis	Deductive framework analysis	Deductive and inductive thematic analysis	Inductive thematic analysis	Framework and narrative analysis	Deductive and inductive thematic analysis

## Findings

We discuss and reflect on two main considerations, which we identified as core features shaping and shaped by the methodological choices we made in the studies we conducted, namely:

1. Team-based approach to rapid qualitative studies.

- Team readiness and expertise.
- Sharing data collection.
- Transcription, summaries, and consequences for analysis.

2. Timely and rapid data collection and analysis.

- Multiple facets of timely data collection.
- Diversity in “rapid” study timeframes.

### Team-based approach to rapid qualitative studies

One of the key features of rapid qualitative approaches is the *extent* to which a team-based approach is adopted. As highlighted by Vindrola-Padros, there is a continuum on which rapid approaches can be placed when it comes to teamwork, ranging from lone researchers to large teams (Vindrola-Padros, 2021a). We discuss here the importance of team readiness and expertise, the practicalities of sharing data collection between different researchers, and what this means for data analysis.

#### Team readiness and expertise

First, the readiness of the team, and familiarity of team members are important to start and conduct a new, or add-on, study rapidly. We found that teams that were already established, or where members already knew each other, were more easily able to rapidly set up and conduct their study. For example, the STEP-UP study was conducted within a larger multi-workstream research program which had started about 4 years before this qualitative study. The team was well-established and familiar, having worked together on different studies, sub-studies, and journal articles. There were several benefits to this, in particular: there was no extra time or effort needed to get to know each person's approach and working style; team roles and responsibilities were already established, meaning that everyone immediately knew what to do and what others were doing; processes for, and approaches to, study set-up and conduct, data collection and analysis, data management and team meetings were already established, allowing a quick and smooth execution.

In contrast, new teams were rapidly assembled in our other studies. For example, in the “HCP Policy and Experiences” study, a new team was set-up including collaborators who had not worked together before, and were from different institutions and research traditions/backgrounds (e.g., health psychology, anthropology, history, clinical medicine). This interdisciplinary collaboration provided much-needed breadth in perspectives on the health crisis, enabling us to identify unique insights and speak to wider audiences. Further, collaborating across institutions meant pooling resources and expertise, and established important new links and relationships. This also enabled producing different types of outputs, including academic journal articles, written evidence submissions to public inquiries, and disseminating findings to policymakers. Nevertheless, our newly-formed interdisciplinary and inter-institutional collaboration also posed some challenges. For example, the rapid and urgent nature of this study meant that, in the initial stages, there was little time for the team to come together to fully figure out how to leverage the benefits of interdisciplinary working more fully, which was consolidated more as the study progressed.

Second, an important part of the team readiness is whether the wider (institutional) infrastructure supports the rapid set-up of studies, with ethical approvals being a key element. For example, in the STEP-UP and SCIENTIST studies, we applied for ethics approvals to amend existing study protocols to address additional research questions and include additional participants. As opposed to designing and approving a completely new study, adapting an existing study enabled a quicker study set-up, participant recruitment, and utilized the resources that were already in place (such as staff/time, funds). In contrast, in the RECOVER-QUAL study, we were able to obtain very rapid ethical approval in some countries but the time to obtain local approvals varied considerably (7–67 days).

Finally, the expertise of team members in terms of qualitative methods is important. In four of our studies, data collection was done by experienced qualitative researchers who each had expertise in conducting interviews with various participant groups. For these projects, we therefore did not often face the task of having to train junior researchers in the basic principles of qualitative research. However, in the RECOVER-QUAL and HOUSEHOLD studies, data collection was shared between interviewers from different countries. Each country led their own data collection, but with the same topic guide being implemented. Due to time pressures, the RECOVER-QUAL core research team prioritized training to all interviewers which focused on understanding the study aims and the topic guide, rather than how to conduct interviews. However, this was complemented by on-demand support for each, depending how much experience they had previously had with qualitative research. In the HOUSEHOLD study, we had one senior colleague providing significant hands-on support and training to an inexperienced qualitative researcher, both in relation to the study aims and the interviewing technique. This was challenging given the tight timelines for the study but the one-on-one training was personalized to meet the needs of the less experienced researcher.

In contrast, across the majority of studies we had limited opportunity to involve other researchers, including more junior colleagues. We therefore did not have a chance to share the workload or speed up the data collection process. This was mainly related to how our research team, consisting of a few experienced qualitative researchers, operated before the pandemic.

Whilst our study teams had extensive expertise in qualitative methods, we were relatively new to the *rapid* qualitative methods. Rapid approaches were determined by the research questions being answered and, as such, we learned more about them through training, engagement with literature and extensive experience when conducting the studies. This involved not only learning the practicalities of conducting rapid data collection or analysis, but also being pushed to quickly examine our own assumptions of whether we believed rapid techniques were credible to us. While for some of the studies, wider study teams included members who have used rapid approaches before, we were not able to fully benefit from their expertise because of rapid timelines. Applying a rapid approach became easier, or more natural, the more studies we worked on, as we started becoming more confident in making, and assessing the consequences of, methodological decisions.

#### Sharing data collection

The fieldwork across all six studies was conducted by teams ranging in size; three studies had the fieldwork conducted by solo researchers, and three studies shared data collection between two or more researchers. In the SCIENTIST, HOUSEHOLD and RECOVER-QUAL studies data collection was shared between two or more interviewers, each conducting interviews in their native language. Sharing data collection had several advantages. First, it allowed sharing workload between the researchers, which in the context of rapid timelines was particularly beneficial. Secondly, using teams in multiple countries allowed us to access participants we would not be able to recruit otherwise. In addition, it enabled us to collect data in participants' native language, thus allowing them to express their thoughts more freely. Thirdly, given the specific context of the COVID-19 pandemic, it was useful to have researchers not only speaking the native language but also understanding the context of each country, specifically relevant COVID-19 policies, current affairs and legislation (e.g., related to quarantines).

In contrast, in the STEP-UP, FACTS and (vast majority of) “HCP Policy and Experiences” studies data was collected by solo researchers. Having a single researcher collecting data was beneficial in particular for the “HCP Policy and Experiences” study, as it enabled the same researcher to build relationships with participants over time. This was important in retaining participants and collecting consistent data across the course of the study. Given that the researcher knew the participants and data so well, this also facilitated rapid analysis and dissemination of findings *via* journal articles led by the same researcher.

### Transcription, summaries, and consequences for analysis

One of the strategies used in rapid qualitative research to speed-up the analysis and/or save cost is to not transcribe the audio recordings of interviews or focus groups, and analyse fieldnotes and/or recordings directly. Out of our six studies, the data were fully transcribed in three, not transcribed in two, and partially transcribed in one. The decision to transcribe or not was dependent on the study aims, timescale and resources, and had important downstream impacts on sharing workloads and the kinds of analysis possible.

For example, in the SCIENTIST, RECOVER-QUAL and STEP-UP studies we were able to secure the resources to transcribe all interviews and rely on transcription to analyse data. The reasons for transcription were slightly different across these studies; the datasets for the RECOVER-QUAL and STEP-UP studies were always planned to be transcribed as the studies were not initially planned to be rapid. In the STEP-UP study, having all transcripts and interview notes also helped another researcher contribute to the rapid analysis as they could quickly and easily familiarize themselves with and code the data. Conversely, in the SCIENTIST study, once we started collecting the data, it became apparent that transcription would be very beneficial as the research team had limited experience of the study topic and data was richer than we initially expected. Here, having access to transcripts allowed us to get a greater understanding of the issues faced by the participants in a shorter amount of time than if we had only had recordings, while also allowing the in-depth analysis to be achieved more quickly.

In the SCIENTIST and RECOVER-QUAL studies, data was charted against a priori categories identified based on the topic guide (deductive analysis) to shorten the time needed for the analysis. However, data within each category was then coded inductively line-by-line to create sub-categories, and identify themes while ensuring that our analysis was grounded in data. Prior to transcription both RECOVER-QUAL and the SCIENTIST study collected interview summaries after each interview or batch of interviews. This enabled the research team to access data quickly prior to it being transcribed and translated into English but also enabled each interviewer to highlight key points from interviews to inform analysis from an early stage. Interview summaries were complemented by discussions within the whole research team to allow interviewers to explain the data collected in the context of what was happening with the COVID pandemic in their own country.

In contrast, the HOUSEHOLD study was set up from the beginning to rapidly inform policy. Similarly, the FACTS study aimed to provide rapid qualitative results to support quantitative findings. As such, these two studies were the most rapid in our portfolio. Transcription was not carried out and this was seen as crucial in speeding up the analysis and the dissemination of results. This impacted the analysis; after each interview, we charted the data onto an a priori framework, including relevant quotes, and discussed the data with other researchers (if applicable). This was a very structured approach, allowing the team to quickly have an overview of the whole dataset. It was also possible as the studies had clear and contained research questions, with datasets analyzed with this lens in mind. This approach contrasted with the interview summaries collected in the studies above which were unstructured and led by each interviewer identifying what they thought was the key information. We felt that the less-structured initial approach was possible as we could still rely on more “traditional” qualitative analysis as a result of access to the transcripts, while the lack of transcripts in the latter studies “forced” us to be more driven by the pre-existing categories to ensure systematic approach to analysis.

Finally, in the “HCP Policy and Experiences” study, notes from all 105 interviews were made by the interviewer summarizing the key points discussed, and case summaries were produced giving an overview of each participants' longitudinal experiences and narrative. Due to resource constraints, only a sub-set (73) of interviews were transcribed. The interviews which were considered particularly important or detailed were selected for transcription. This pragmatic approach had benefits as well as challenges. While transcripts are important, they are not the only source of data in an interview study. Keeping fieldnotes alongside interviews was helpful in capturing aspects of the interviews that were not necessarily captured by transcribing what was said; they also allowed capturing communication occurring before or after the recorder was turned on/off, or through other mediums (e.g., email). On the other hand, the verbatim transcripts provided a detailed record of the content of the interviews, and thus allowed for thematic coding and analysis of the data. Unstructured notes were helpful to inform interpretation, whereas time pressure meant that re-listening to all recordings was often unfeasible. These considerations became particularly pertinent when a new researcher joined the team to conduct further analysis of these data. This second researcher was less familiar with the interviews that were not transcribed, and while the notes helped give a rapid introduction and overview of the dataset, they found the verbatim transcripts particularly helpful. Therefore, when working with transcribed and non-transcribed data, and sharing data analysis with a researcher who did not collect the data themselves, there was a tendency to give more attention to transcribed interviews because they could be more easily coded and quoted.

### Timely and rapid data collection

Timely and rapid data collection are two important features of rapid research; data needs to be collected quickly and at informative timepoint(s). We discuss here ways in which timeliness and rapidity became pertinent to our studies.

#### Multiple facets of timely data collection

Rapid research is often considered as research conducted within a short time, although the duration of the rapid studies also differs largely. Rather than focusing just on the overall timeline or duration of the study, we found considering the timeliness of the data collection a key and helpful aspect of rapid research. When conducting our studies, we became aware of the multidimensionality of the concept of timeliness. Here we discuss three aspects related to timeliness of data collection: (i) capturing the phenomena of interest in real time; (ii) complexities of mixed-methods studies; and (iii) ever-changing context of pandemics.

##### Capturing the phenomena of interest in real time

Perhaps the most obvious dimension of timeliness is whether the data is being collected in a way that allows researchers to capture phenomena of interest in *real* time. Although the benefit of “hindsight” can be of particular significance, gathering data as things are happening, rather than retrospectively, has great advantages especially when needed to inform policy and emergency responses. It allows exploration of issues as events unfold, and uniquely captures participants' insights, views, and sense making in the midst of their experiences prior to subsequent reflections and reinterpretations.

In the RECOVER-QUAL study, we were able to interview HCPs in the first few weeks of the pandemic (the first lockdown). As we were interested in how they were adjusting to the changes in care delivery, they could describe these changes almost as they were happening. Some participants commented how even a week could make a difference in how they felt about the situation, as it was changing very rapidly on the ground. In contrast, we interviewed some participants in later months, but still within the period of the first lockdown. This “delay” was due to ethical approvals taking longer in some countries. These later interviews were slightly different as participants had more time to adjust to the changes in primary care and, importantly, to process what was happening and how they felt about it. This meant that the interviews were to some extent retrospective and participants often described how they felt initially and how they felt at the time of the interview.

In addition, the aim of the “HCP Policy and Experiences” study was to explore the experiences of HCPs during the COVID-19 pandemic, and how they changed over time. The first pilot interviews were conducted in February 2020 at the very start of the pandemic in the UK. To rapidly start the study and capture experiences “in real time,” participants were recruited through contacts/networks of the research team members. While this strategy enabled a prompt start and recruitment, recruiting a wider range of participants and purposeful sampling were more difficult. In this longitudinal study, participants were interviewed between 4 and 10 times throughout the first year of the COVID-19 pandemic. Interviews were scheduled depending on participants' availability and the pace of changes in their work (e.g., roles and responsibilities), guidelines and the pandemic's impact on healthcare services. This allowed us to collect timely (“real-time”) data, which could identify trajectories of how HCPs' experiences changed over time throughout a rapidly-changing context.

In contrast, in our STEP-UP study, we wanted to capture the impact of the pandemic on antibiotic prescribing and stewardship. However, we were reluctant to add burden and additional pressure on HCPs to participate in the study early in the pandemic when clinicians had other priorities. When we conducted the interviews in autumn 2020, we found that HCPs perceived their antibiotic prescribing as elevated early in the pandemic, and then returning to more usual in autumn. Although conducting the interviews later in the pandemic meant that we did not capture the perceived impact in “real time,” we were able to explore HCPs' reflections of the few months at the time when they seemed in a better position to reflect and share their experiences.

##### Complexities of mixed-methods studies

Mixed methods research often poses challenges in integrating datasets. In the context of rapid research, this alignment between the timeliness of data collection and integrating datasets became even more important. Two of our studies, the FACTS and HOUSEHOLD studies, were qualitative studies conducted alongside quantitative components, thus making timeliness of data collection of the two components closely related.

The FACTS study was a cohort study with a qualitative sub-study. The cohort study ran from October 2020 to January 2021, and aimed to examine the feasibility of regular self-testing for SARS-CoV-2 using LFTs in a university setting (Hirst et al., 2021). To complement this work, we conducted a qualitative study looking at acceptability of the testing, by doing interviews with university students and staff. To ensure consistency in timeframes for both studies, data collection for the qualitative study had to be completed within the timeframe of the cohort study. Specifically, we wanted to avoid interviewing people about their experiences of using LFTs beyond the period of the cohort study to ensure that interview participants had not had significantly greater experience of self-testing. Similarly, in the HOUSEHOLD study it was crucial that we were able to conduct interviews with participants while they were still in quarantine to capture how their experiences of adhering to infection control measures unfolded. We conducted interviews 7–15 days after the COVID-19 diagnosis of the index case, but this required a great time commitment by both researchers working on the project and close collaboration with the team recruiting patients in practice.

##### Ever changing context of pandemics

Finally, the context of the pandemic became very important in examining whether data was collected and disseminated in a timely manner. This context—shaped by local guidance and (inter)national public health policies such as those related to testing, quarantine requirements, and models of delivery in and access to primary and secondary care—became central for us to understand in order to interpret participants' experiences.

In the HOUSEHOLD study, context became particularly pertinent to timely data collection. Specifically, as the study was conducted in Belgium and the Netherlands, we became acutely aware of the significance of the policy changes relevant to the study aims. Even though the study was being conducted at the same time in the two countries, the COVID-19 restrictions and regulations related to quarantine requirements in both countries were changing rapidly. This influenced interpretations of participants' views on quarantine and infection control measures. As a result, we allocated extra resources to collect data in both countries as closely as possible to each other. We also closely monitored changing guidelines to be ready to consider, albeit often at short notice, what it might mean for the data collection.

In the “HCPs Policy and Experiences” study, we faced similar issues, especially as one of the aims of the study was to explore the impact of the changing COVID-19-related policies and guidelines for HCPs. We also included participants from different settings (general practice, emergency care, different hospital departments) where policies and practices often differed, and changed frequently, so we had to keep track of a vast number of contextual and policy shifts. We did this through linking policy or guidance documents to international monitoring of key policy and guidelines available online, and keeping a record of guideline documents and announcements (including clinical practice, infection prevention and control, public health, and occupational health and safety guidelines); particularly those related to any changes mentioned by participants in interviews. Although this added a large amount of additional work, this was especially helpful in informing our analysis. We could contextualize our year-long longitudinal data against a policy timeline of relevant guidelines and guideline changes that we constructed from tracking these in real-time.

Finally, working with policy colleagues also allowed dissemination of findings in a timely manner, in relation to the ever-changing policy landscape. Therefore, in addition to traditional dissemination channels such as scientific publications, for three of the projects (HCP Policy and Experiences, HOUSEHOLD and RECOVER-QUAL) we worked closely with policy partners to disseminate the findings in the form of policy briefs, summaries, or regular updates to policymakers (e.g., European Centre for Disease Prevention Control, 2020; World Health Organisation, 2020). Regardless of the overall study timeframes, we were thus able to rapidly disseminate findings to different audiences as data collection was still ongoing.

#### Diversity of “Rapid” study timeframes

Our studies ranged in timeframes, from days to a few months, with the longest, a longitudinal study, conducted over a year. Drawing on terminology from longitudinal research, we consider the study *timeframe* (period over which the data is collected) and the *tempo* (intensity) of data collection in tandem, to reflect on what “rapid” meant in our studies.

##### Rapid timeframe and intensive tempo

In our FACTS study, we faced a particularly rapid timeframe, which was planned for only 2 months (December 2020–January 2021). However, the study frame was shortened even more as the study had to pause for 2 weeks when university students and staff went on their Christmas break. This meant that recruitment and data collection had to be particularly condensed which resulted in 18 interviews being conducted across just 13 days, with many instances of interviews being conducted one after another. While we successfully completed data collection within this timeframe, it required significant re-organization of workload within the team related to other studies being conducted at the same time. The data collection and analysis were conducted by one person which put a particular pressure on the timely delivery. A team-based approach to data collection might have been particularly useful here to share this intense fieldwork.

In contrast, the SCIENTIST study and the RECOVER-QUAL studies had different timeframes and tempos. The data collection timeframe for the SCIENTIST study was 5 months, with 21 participants. While we collected and analyzed data simultaneously, thus allowing for a rapid dissemination, the tempo of data collection was slower as it largely depended on access to and availability of the participants (scientists working on the COVID-19 advisory boards). Similarly, the RECOVER-QUAL study had a 4-month data collection timeframe, but the tempo of data collection in each country was more intense (2–6 weeks) to reduce diversity in experience within countries.

##### Longitudinal design: The case for a longer timeframe with intensive tempo

Although a longitudinal design might at first seem contradictory to rapid research, based on our reflections from conducting two longitudinal studies (STEP-UP and “HCP Policy and Experiences” study), we examine how a longer timeframe may be employed together with a more intensive tempo of data collection, analysis and dissemination.

The “HCP Policy and Experiences” study was designed from the outset as a longitudinal qualitative study that aimed to follow HCPs over a year and explore how the context (e.g., policies, guidelines) and their experiences changed over time during the pandemic. The value of a longitudinal design is that it allows researchers to explore what changes, or does not change, over time through multiple data collection points with (usually) a smaller sample of participants. The timeframe (in our example—a year) might not as such match the typical shorter timeframes of rapid research. However, the tempo of data collection and analysis was intensified and enabled by using some rapid research techniques. The time for approvals, set-up and recruitment were shortened and intensified (compared to standard qualitative studies) by prioritizing resources and the study for approvals, and by recruiting participants through existing networks. Data collection was also intensified as we started collecting interviews as soon as participants were identified in the early stages of the emerging pandemic, and we arranged frequent interviews (depending on each participant's availability) over the first months of the pandemic when policy changes occurred rapidly. Finally, data was analyzed alongside data collection, with ongoing dissemination of the emerging findings on a weekly basis in the form of updates to policymakers, and preparing academic publications at points throughout data collection.

## Discussion

In this manuscript, we described the most salient methodological issues that we faced when setting up and implementing six rapid qualitative studies during the COVID-19 pandemic. As others have highlighted, there is a need to openly discuss methodological choices in rapid research, to promote transparency in reporting, assist other researchers in making informed choices, and consequently move the field forward (Vindrola-Padros, 2021a). Here, we reflected on two interconnected issues, often central to rapid qualitative approaches. We also provide a summary of key considerations in relation to discussed methodological dilemmas in Table 2.

**Table 2:** Summary of key considerations in relation to methodological choices.

**Methodological choices**	**Key considerations**
Implementing a team-based approach vs. a solo researcher approach	• The breath of the research question (narrow or broad, exploratory questions) • The need to maintain rapport and minimize sample attrition (e.g., in a longitudinal study) • The tempo of data collection and analysis (e.g., more or less intensive) • Complexity of data collection (e.g., involving multiple settings, countries, or topics) • The need to invest time and training in larger teams, bringing together divergent viewpoints and methodological expertise • The novelty of the topic to the research team • Structure of the research team and ways of working (e.g., clear roles and responsibilities, regular meetings and updates)
Transcription of data	• The type of analytic approach (e.g., structured/deductive analysis or inductive analysis) • Priorities, and constraints of the research project (e.g., money, time, policy relevance, engagement with local stakeholders) • The size of the research team and which researchers are analyzing the data • The extent to which researchers use field notes, summaries and group discussions to support the process of making sense of the data
Conducting timely research	• (Changing) external context of the study • The need to invest resources to be able to collect data in *real* time • Complexity of design (stand-alone or mixed methods studies) • Workloads of potential participants and ethical responsibility in collecting and not collecting data in *real* time
Conducting rapid research	• The required timeframe (period of data collection) and tempo (intensity of data collection) • Availability of staff to ensure rapid data collection and share workload

### Considering how to ensure a suitable and successful team-based approach to rapid research

A team-based approach is one of the key features of rapid qualitative research. While some consider it essential (McNall and Foster-Fishman, 2007), others suggest considering a team-based approach on a continuum from solo researchers to larger teams, depending on the study design (Vindrola-Padros, 2021a). In our studies, we utilized both a solo-researcher and a team-based approach to data collection and analysis. It is important to reflect which of these may be most suitable for a study, and researchers may want to take into account a number of factors. Firstly, one of the key considerations might be the tempo and complexity of data collection and analysis. We found a team-based approach most beneficial in studies with a more intense tempo and more complex data collection (e.g., involving multiple countries, settings, topics/research questions). As also discussed by others, larger research groups were hugely valuable in enabling workload-sharing, better access to participants, faster data collection, collection of data in local languages, and allowing the team to benefit from insights related to local contexts when collecting and interpreting the data (Graetz et al., 2022). However, as others highlighted as well (Vindrola-Padros et al., 2020), larger research groups pose the challenge of ensuring a shared understanding of the methodological approach to qualitative research being undertaken. Related to that, it may be difficult to bring together potentially divergent viewpoints of researchers coming from different disciplines and traditions (Vindrola-Padros and Johnson, 2020). While this can be offset by investing time in appropriate training and collaborative team meetings, the larger the team, the more difficult it may be to do that. The challenges of interdisciplinary research are well-established (Larsen, 2018; Bardosh et al., 2020), but working under tight timelines, in newly established teams can magnify these challenges (Baxter et al., 2021; Colman et al., 2021). Given the great value of interdisciplinary working, especially in healthcare emergencies, practical strategies may help to manage some of the challenges we experienced; for example, using Rapid Assessment Process (RAP) sheets to facilitate more systematic updates, summaries of data and a more systemic approach to building infrastructure for cross-country and/or interdisciplinary research (Vindrola-Padros, 2021a). Secondly, the team readiness, and related to that, the novelty of the topic to the study team, may also be important. In some of our studies we benefited from being able to work with researchers who we knew well and had experience of using qualitative methods. We also found that when the topic was new to (some of) the research team, it was also useful to adopt a team-based approach to share insights, and leverage individuals' expertise. Others have also highlighted that a team-based approach can be a good way of sharing existing expertise and having a lead researcher familiar with the topic, can be useful in ensuring that the rest of the team can contribute to the analysis (Vindrola-Padros et al., 2020). Thirdly, the scope of the study and breath of research questions can also be an important consideration. We found that a solo researcher approach was most beneficial for studies with narrower research questions (i.e., rather than broader, more exploratory ones), which rely less on team input for data collection and analysis. Finally, for the longitudinal studies, in line with other researchers (Worth et al., 2009), we found that one person collecting all data facilitated rapport and relationship-building with the participants. While in the rapid studies, this may not always be seen as a priority, it is an important consideration to ensure low sample attrition. Thus, we would urge researchers to carefully consider the suitability and implications of team-based vs. solo approaches. Particularly, the potential trade-offs involved as well as the provisions necessary to support the approach taken, make it an effective use of resources, and derive the most benefit from it.

### Considering benefits and challenges related to transcribing data

The traditional approach in qualitative research often involves audio recordings and transcription, with the aim of using the transcripts for analysis (Greenwood et al., 2017). Transcription has often been thought of as a non-negotiable part of qualitative interview research, and challenging this can be difficult (Vindrola-Padros and Johnson, 2020). However, some have highlighted the importance of considering the diversity of qualitative traditions and schools, and that while transcription can be of great value to some qualitative approaches, for others it may not be essential (Halcomb and Davidson, 2006). Rapid studies may in particular eliminate transcription of data, and thus it is important to consider both the suitability of (lack) of transcription in this context, as well as the downstream consequences including workloads and the type of analysis that is possible. Firstly, as highlighted by others, transcription decisions need to be closely linked, and appropriate, to the study aims and analytic approach (Tessier, 2012). In line with others (Gravois et al., 1992; Halcomb and Davidson, 2006), we felt that transcription provided more flexibility during thematic analysis as it facilitated making conceptual links between categories. Transcription may be even more important and beneficial for qualitative approaches which rely on making these conceptual links in order to develop theory, for example in grounded theory (Walker and Myrick, 2006). There is a paucity of rapid qualitative research involving such qualitative methodologies, and the studies published during the pandemic using grounded theory seem to rely on transcribed data to be able to create conceptual frameworks based on the results (e.g., Rees et al., 2021; Hörold et al., 2022). This is perhaps not surprising as conceptual analysis or drawing on theory takes time which may not be always compatible with rapid research timeframes (Vindrola et al., 2021a). Thus, while it may be difficult to implement a grounded theory methodology in a rapid study, it is important to highlight that researchers using such methodologies, may choose to adopt a discrete rapid technique at different stages of data collection or analysis, if their aim is to reduce the time required for data collection or analysis for these parts of the research process. It is also worth noting though that even when having access to transcripts, fieldnotes collected during or after interviews, and interview summaries, are also greatly beneficial in making sense of the data. Fieldnotes have a long standing place in qualitative research and can add an important layer to the analysis (Phillippi and Lauderdale, 2017), as transcripts cannot be assumed to be the only source of data in an interview. In contrast, in our studies which relied on more descriptive analysis, the lack of transcription was not disadvantageous. Thus, more descriptive analysis was possible based on recordings only, but the availability of transcripts further facilitated making links between categories. Secondly, the researchers may want to reflect on whether their motivation for omitting transcription is to save time or money. Specifically, researchers seeking to save money may want to omit transcription but then aim to “counterbalance” the lack of it by committing (significant) time to formulating codes and themes based on extensive listening to audio recordings (Gravois et al., 1992; Greenwood et al., 2017), or introduce an additional step in data collection where researchers create a mind map with participants in a focus group, which would be an equivalent of generating of “codes” or “categories” (Burgess-Allen and Owen-Smith, 2010). In the context of the healthcare emergency such as the COVID-19 pandemic, saving time and rapidly analyzing data, may be the most important motivator (Johnson and Vindrola-Padros, 2017; Vindrola-Padros et al., 2020; Hoernke et al., 2021). In this instance, researchers may omit the transcripts and analyse the data directly from the recording, which may involve producing a list of initial issues (themes) after each focus group/research encounter that are then ranked later on (Joe et al., 2015), or using RAP sheets (Vindrola-Padros, 2021a) in order to speed up the process. In our studies we were often focused on producing actionable results, and were motivated by the aim of influencing policy based on incoming data. Thus, regardless of whether the transcription was possible or not, we relied on more descriptive and structured analysis to formulate a reply to a focused research question. Thirdly, it is worth considering who will conduct the analysis and how transcription may affect this process. In our studies, transcription of data allowed researchers who did not collect the data to more quickly familiarize themselves with the data and contribute to the analysis, and it made it easier to select supporting/illustrative quotes when writing up. Related to that, it is worth reflecting on the need for a transparent and permanent record of the data collected and analysis, particularly for studies with richer datasets and/or with additional research questions for future secondary analyses. Overall, given the variety of approaches possible with and without transcripts, we urge researchers to be clear about their priorities (e.g., time, cost, impact) as these have important implications for the type of analysis possible and/or appropriate. To support researchers in making such informed choices and ensuring study quality, sufficient training and expertise specifically in employing rapid qualitative approaches should also be sought (Vindrola-Padros, 2021a).

### Considering how to ensure timely research

Rapid research is often motivated by the need to be responsive to changing priorities, thus ensuring its timeliness (Vindrola-Padros, 2021b; Vindrola-Padros et al., 2021). Timeliness of research has been somewhat discussed in the literature, mainly in relation to evaluations, with authors highlighting that *when* the research is conducted is as important as whether it addresses the “right” issues (Grasso, 2003; McNall et al., 2004). For healthcare research to be useful, its findings need to be rapid, responsive, and relevant (Riley et al., 2013). In the context of health emergencies, Vindrola-Padros et al. (2020) also highlighted the importance of research timeliness and its ability to deliver timely and actionable findings, which can inform evidence-based public health response. However, for social scientists, including qualitative researchers, an important aspect of timeliness is that it is partially dependent on whether these researchers are invited to contribute to the pandemic response early enough (Vindrola-Padros et al., 2020). Thus, the discussion around timelines has been focused mainly around whether the study findings are produced in timely way, so they could inform the policy decisions, or at least contribute to the evidence being considered (Grasso, 2003; McNall et al., 2004). While these are essential features of rapid research, the COVID-19 pandemic has brought out another aspect of timeliness related to when the data was collected, rather than only to when it was used. Specifically, in the rapidly changing context of COVID-19, our studies highlighted the importance of three additional aspects of timeliness: collecting data in real time (rather than retrospectively), carefully considering the changing external context, and the complexities of mixed methods studies. These aspects have been discussed to a lesser extent in the methodology-focused literature.

Timeliness of findings is of course closely linked to timely data collection, but it perhaps has not been acknowledged to the same extent (with some exceptions, e.g., Vindrola-Padros et al., 2020). We have illustrated here that timeliness is a distinctive feature of rapid research. Thus, it is possible to have timely findings, for example through simultaneous data collection and analysis, but still not collect data in real time. Hoernke et al. (2021) also highlighted this issue as they collected interviews with HCPs before, during and after the first peak of the pandemic, with authors noting that this approach allowed them to capture HCPs' experiences as the situation was unfolding. When attempting to collect data in real time, researchers may want to consider the feasibility of such an approach. In our studies, we have discussed the importance of considering the extent of heterogeneity between the countries collecting data within the same study. In studies conducted in multiple settings or countries, it is important to reflect on and identify the key differences between these settings or countries which may impact how researchers interpret the data, especially if the periods of data collection are not aligned. Others have acknowledged the complexities of implementing studies in multiple countries during the COVID-19 pandemic, and the limitations of not gathering comparable data (Ding et al., 2021; Kilian et al., 2021, 2022); however, these aspects have not been highlighted as an important dimension of timelines in rapid qualitative research across multiple sites. Nevertheless, there remains the need to consider the burden and additional pressure on participants taking part in the studies in real time (Vindrola-Padros et al., 2021). Our study highlights that researchers should reflect on the opportunities and costs offered by gathering data in real time, and its impact on participants. Finally, the diversity of designs of qualitative and mixed methods approaches have been highlighted before (Vindrola-Padros, 2021a), including conducting (i) a rapid study to inform longer-term research, (ii) a shorter study exploring remaining questions after a longer study has been completed, or (iii) a parallel rapid study to a longer program of work. Conducting mixed methods research is challenging as it requires an integration of research teams conducting each sub-study, as well as a clear strategy for triangulating the data (Tashakkori and Creswell, 2007; O'Cathain et al., 2010). Our studies conducted during the COVID-19 pandemic also highlight an additional challenge for certain mixed-methods designs, namely the need to align the data collection timelines to ensure that the data is comparable and can be truly triangulated. This also requires careful planning and appropriate resources.

### Considering how to ensure rapid data collection

Rapid timeframes, understandably, are considered a key feature in rapid qualitative approaches. We have found that a useful way of considering the extent to which the study can be considered rapid is not only timeframe of data collection but also its tempo. Both terms have a long-standing use in longitudinal qualitative research. While a timeframe can be understood as the length of data collection, tempo can be defined as the number, length and frequency of visits to the field (Neale, 2021). While the frequency of visits is of course a unique feature of the longitudinal design, the number and length can be particularly useful when considering the rapid qualitative research as well. This has implications for how we define what rapid is; while it may be difficult to define the study length for the study to be classed as rapid because the extent of rapidness will depend on the aims, research question, context and other factors, there are also attempts to create a boundary with some suggesting that data collection should not exceed 6 months. This is on the basis that data collection longer than that will start resemble a non-rapid study (Vindrola-Padros, 2021a). Interestingly, similar arguments have been expressed in relation to longitudinal qualitative research, highlighting that there is no universal length of data collection period, as this will greatly depend on the study objectives. For example, Saldana coined the term “shortitudinal,” to describe studies which combine intensive data collection periods with shorter time frames (starting from several months) (Saldaña, 2003). On a practical level, the researchers may want to consider the tempo of their data collection. For example, a 4-month study with 80 interviews (as for example in the RECOVER-QUAL study) may demand different approaches and resources than a 5-month study with 21 interviews (as was the case with our SCIENTIST study). Thus, the required resources, staff workloads and competing priorities across multiple projects, and the type of analysis will have to be considered. Studies utilizing more intense tempo, may benefit from a team-based approach to manage workloads. However, even a team-based approach may not allow a more conceptual analysis in short periods of time, and thus more structured approach may need to be considered.

A particular example of the tension and importance of considering both timeframe and tempo might be a longitudinal design in the context of rapid research. At first glance, longitudinal design and rapid research seem incompatible. As highlighted earlier, this closely links with an idea that rapid studies are conducted over relatively shorter periods of time, and thus not allowing space (and time) for dealing with challenges related to more complex designs. In the field of rapid qualitative research, longer timeframes have been somewhat indirectly discussed in relation to some rapid qualitative approaches such as Rapid Feedback Evaluation or Rapid Cycle Evaluation. These approaches are considered as either having short study timeframes or having longer timeframes with built-in feedback loops/cycles for the continuous sharing of findings (Vindrola-Padros et al., 2021), with the latter potentially making the studies longer overall. The context of the pandemic also puts these “traditional” timeframes in spotlight. Despite a great number of qualitative studies examining experiences of patients and HCPs during the pandemic, longitudinal rapid qualitative design has been utilized less frequently. This is not surprising; longitudinal design is still underutilized in applied healthcare research (Wanat et al., 2021d). However, there are notable examples of combining longitudinal design and rapid research; for example a study by Turner and colleagues who examined how GP practices maintained face to face contact by conducting four interviews between May and June 2020 through combining rapid timeframes and timely dissemination, with longitudinal design (Turner et al., 2021). It is also worth noting that, similarly to grounded theory studies conducted during the pandemic discussed earlier, the studies which used longitudinal designs and were conducted over a short period of time have not always been classified as rapid by the authors themselves (e.g., Maison et al., 2021). This highlights that short data collection period does not automatically lead to a study being called “rapid.” It also shows the complexities in defining the key characteristics of rapid studies, and applying these when designing and implementing rapid qualitative approaches. Based on the recent examples, there seems to be a scope for innovation in rapid qualitative researchers by adopting more complex designs with both shorter and longer study timeframes.

## Conclusions

Rapid qualitative research can be successfully set up and implemented in the context of a healthcare emergency, but can pose methodological dilemmas and challenges for researchers. In this manuscript, we have focused on two methodological issues, which became pertinent to our studies, namely implementing a team-based approach, and conducting timely and rapid research. By sharing our experiences and reflections, we hope to contribute to the transparency in conducting and reporting rapid studies and help other researchers to make better informed methodological choices. We also encourage other researchers in engaging with such methodological discussions to help move the field of rapid qualitative research forward.

## Data availability statement

The original contributions presented in the study are included in the article. Further inquiries can be directed to the corresponding author.

## Ethics statement

The studies involving human participants were reviewed and approved by relevant Ethics Committees. RECOVER-QUAL—Ethical approval for the whole project was granted in England by the South Central Berkshire Research Ethics Committee (reference number: 20/SC/0175). The seven research sites outside of the UK also obtained ethical approval from their local organizations. FACTS—The study was approved by the University of Oxford Research Ethics Committee in October 2020 (CUREC ethics reference R72896/RE001). SCIENTIST—The study received ethical approval from the Ethics Committee of Antwerp University Hospital (20/13/150). HOUSEHOLD—The study received ethical approval from the Medical Ethical Committee Utrecht (NL) and Medical Ethics Committee UZA Antwerp (BE) (Reference number 20-185/D and 20/14/ 177 respectively). STEP-UP—The study was reviewed and approved by the University of Oxford Medical Sciences Inter-Divisional Research Ethics Committee (ref. R59812) and the NHS Health Research Authority (ref. 19/HRA/0434). HCP Policy and Experiences—The study was approved by the University of Oxford's Medical Sciences Interdivisional Research Ethics Committee (ref. R69302). Participants in all studies gave informed consent to participate in the research, and their consent was documented.

## Author contributions

MW led the writing of the first draft. All authors contributed to writing the first draft of the manuscript. All authors contributed to conception and design of the study, manuscript revision, read, and approved the submitted version.

## Funding

The RECOVER-QUAL, HOUSEHOLD, and SCIENTIST studies were part of the RECOVER (Rapid European COVID-19 Emergency Response research). RECOVER was funded by the EU Horizon 2020 research and innovation program under (Grant Agreement No. 101003589). FACTS study did not receive external funding. STEP-UP study was funded by the Economic and Social Research Council (ESRC) through the Antimicrobial Resistance Cross Council Initiative supported by the seven research councils in partnership with other funders (Grant No. ES/P008232/1). It was also supported by the NIHR HPRU in Healthcare Associated Infections and Antimicrobial Resistance at the University of Oxford and Imperial College London in partnership with UK Health Security Agency, the NIHR Oxford Biomedical Research Centre, and the NIHR under the Applied Health Research (ARC) program for North West London. HCP Policy and Experiences study was funded by the UKRI/NIHR 2019 nCoV Rapid Response Call through a grant (Grant No. NIHR200907) which supported AB and CP, with support from the NIHR HPRU in Emerging and Zoonotic Infections at University of Liverpool in partnership with UK Health Security Agency, and in collaboration with Liverpool School of Tropical Medicine and the University of Oxford. ST-C is funded by the NIHR HPRU in Healthcare Associated Infections and Antimicrobial Resistance at the University of Oxford in partnership with UK Health Security Agency.

## Conflict of interest

The authors declare that the research was conducted in the absence of any commercial or financial relationships that could be construed as a potential conflict of interest.

## Publisher's note

All claims expressed in this article are solely those of the authors and do not necessarily represent those of their affiliated organizations, or those of the publisher, the editors and the reviewers. Any product that may be evaluated in this article, or claim that may be made by its manufacturer, is not guaranteed or endorsed by the publisher.
